# Changing trend of HIV, Syphilis and Hepatitis C among Men Who Have Sex with Men in China

**DOI:** 10.1038/srep31081

**Published:** 2016-08-18

**Authors:** Qianqian Qin, Weiming Tang, Lin Ge, Dongmin Li, Tanmay Mahapatra, Liyan Wang, Wei Guo, Yan Cui, Jiangping Sun

**Affiliations:** 1 National Center for AIDS/STD Control and Prevention, Chinese Center for Disease Control and Prevention, Beijing, China; 2 University of North Carolina at Chapel Hill, Project-China. No. 2 Lujing Road, Guangzhou, 510095, China; 3 School of Medicine, University of North Carolina at Chapel Hill, Chapel Hill, NC, 27514, USA; 4 National Institute of Cholera and Enteric Diseases, Kolkata, 700010, India

## Abstract

Dearth of information regarding the trend and correlates of HIV, syphilis and Hepatitis C (HCV) in a country-wide sample of understudied though high-risk Chinese men who have sex with men (MSM) called for a comprehensive serial cross-sectional study. Using a multistage mixed-method strategy, 171,311 MSM from 107 selected cities/counties in 30 provinces of mainland China, were interviewed and tested. Descriptive, bivariate, multivariate and Cochran-Armitage trend analyses were conducted using SAS 9.2. During 2009-13, recent (71.5% to 78.6%, p < 0.001) and consistent (40.4% to 48.8%, p < 0.001) condom use as well as condom use during commercial anal sex (46.5% to 55.0%, p < 0.001) were increasing. In contrast, commercial anal sex with male (11.9% to 7.1%, p < 0.001) and drug use (1.9% to 0.8%, p < 0.001) were decreasing over time. HIV prevalence increased gradually (5.5% to 7.3%, p < 0.001), while syphilis (9.0% to 6.3%, p < 0.001) and HCV prevalence (1.5% to 0.7%, p < 0.001) decreased over time. A positive correlation was observed between HIV and syphilis prevalence (r = 0.38). HIV infection was associated with HIV-related knowledge, services and injecting drug use. An increasing trend of HIV prevalence was observed during 2009–13 among MSM in China. While gradual reduction of risk behaviors along with syphilis and HCV prevalence supported expansion of testing and prevention services, increasing HIV burden called for deeper thematic investigations.

Despite concerted global efforts for years to contain HIV, especially targeting the at-risk groups, occurrence of new infections among men who have sex with men (MSM) continued to increase in the developing world including Africa, China, Taiwan, Myanmar, India and Thailand^1^,^2^. Although driven historically by certain high-risk groups like injecting drug users and former blood and plasma donors, in recent past, HIV epidemic in China started to reflect diverse epidemiology and transmission patterns^3^,^4^,^5^,^6^,^7^. During 2011, the number of people living with HIV/AIDS (PLWHA) was estimated to be 780,000 in this country, 63.9% of which were infected through sexual transmission^7^. The proportion of PLWHA infected through male-to-male homosexual route did increase here, from 2.5% in 2006 to 17.4% in 2011^7^. Although improvements in the infrastructure and coverage of the National HIV surveillance system in China could partially explain this observed increasing trend, potential reasons for actual rise should also be explored. During 2011, in this country, the estimated size of MSM population was 5–10 million with an HIV prevalence of 6.3%, substantially higher than the corresponding value in the general population (0.058%). These male-to-male homosexually transmitted HIV infections accounted for 29.4% of the estimated 48,000 new infections in 2011, despite implementation of comprehensive control measures for years by Chinese Government^3^,^4^,^6^,^7^,^8^,^9^,^10^. Occurrence of other sexually transmitted infections (STIs), for example syphilis and hepatitis [including hepatitis C (HCV)], were reported to be high among MSM potentially attributed to their high-risk sexual behaviors and lower coverage of preventive measures^8^,^9^,^10^,^11^,^12^,^13^,^14^. Major barriers included social taboos and discrimination, for which Chinese MSM remained mostly hidden^8^,^13^. Majority of the studies conducted among MSM in China till date had limited applications in terms of extrapolation to bigger population, owing to non- representative sampling, methodological inconsistencies and potential vulnerability to systematic errors^13^,^15^.

Established socio-demographic, environmental, behavioral and policy related contextual risk factors for HIV were all found to be largely inter-related. Thus having a deeper understanding of the interplay between such risk factors appeared to be mandatory for further reduction in spread of HIV from and within the Chinese MSM population. Therefore, in order to make a tangible and sustained preventive impact on HIV epidemic among MSM in this country, it was essential to conduct detailed explorations of reliable and accurate behavioral data so that the findings could be translated into effective multilevel public health interventions to combat the epidemic^16^,^17^,^18^.

There was also a pressing need for additional research on changes in the dynamics of HIV and STI epidemics and risk behaviors for providing adequate insight regarding need assessment, program planning, and comprehensive policy response. But studies investigating such trends in prevalence of HIV, other STIs (including syphilis and HCV) and risk behaviors among Chinese MSM were limited.

Thus a serial cross-sectional study was called for to determine the trends of the aforementioned parameters and their interplay involving a multistage sample of MSM that could ensure somewhat national-level representativeness (as much as possible).

## Methods

### Design

A serial cross-sectional study was conducted between 2009 and 2013, involving a sample of MSM population residing in mainland China, to determine the trend of HIV, syphilis and HCV infections among them.

#### Ethics statement

Informed consent was obtained from each participant prior to the interview and blood collection. The study was carried out in “accordance” with the approved guidelines. The study protocol, contents and procedures were reviewed and approved by the Institutional Review Board of the National Center for AIDS Prevention and Control (NCAIDS), Center for Disease Control (CDC), China.

### Recruitment

As a rule of thumb, and based on the recommendation of WHO and experience from other countries^19^,^20^,^21^, a minimum sample size of 250 to 400 participants is recommended for each site^22^. This recommendation is based on the following formula: N = [4*z_α_^2^ *P (1 − P) ]/W^2^. At here, z_α_ is a factor that corresponds to the desired confidence interval (for a 95% confidence level, z_α_ = 1.96); P is the expected proportion of patients with the outcome (such as HIV prevalence, in China, it is between 5–20%, depend on the location of the site); W is the width of the interval, for example the width for a margin of error of +/− 3% is 0.06^20^,^21^.

For participants’ recruitment, in each of the study city/county, the local Center for Disease Control (CDC) at first prepared an exhaustive list as well as map of all the potential venues in the respective site where MSM were usually found to cruise or MSM activities were known/speculated to take place. Then in each of these venues, personnel from CDC accompanied by peer outreach workers paid visits every day for one week and recorded the daily attendance size of the respective venue during that week. For the probability proportional to size sampling used in this research, the size of attendance of MSM in each of these sites was then determined by calculating the mean number of participants visiting that particular venue per day.

A multistage mixed method sampling strategy was used, involving venue and internet based sampling, in addition to snowballing. Based on the sample size calculation, sampling was conducted in 107 city/county level regions in 30 provinces, municipalities and autonomous regions of Mainland China (except Tibet). While applying the sampling design, appropriate sampling frame was obviously not available, because of the largely hidden nature of the MSM population in China. Hence we had to apply snowballing: a sampling strategy specifically designed for sampling a hard-to-reach, hidden population. But snowballing alone was not adequate for recruiting a nationally representative sample owing to the generally non-random selection of the initial subjects and the probability of larger networks being over-represented. Hence venue-based (using probability proportional to size for selection) and internet-based sampling were also conducted additionally. In each of the study sites, same sampling design and study protocol were followed and staffs were trained as per the same training module.

Adult (aged 18 years or more) men, who had sex with men during the past year and provided informed consent in favor of participation, were eligible for the study. Among the eligible subjects, those who already participated in this survey in the same or other city/county during the same year were excluded.

For venue based sampling, in each selected site (city/county), with the help of community based organizations (CBOs), all known venues were mapped and categorized into two groups. Pubs, discos, tearooms, clubs, bathhouses, saunas and massage parlors were classified as Group A while parks, public restrooms and public lawns were categorized as Group B venues. From both the groups, based on the size of attendance of MSM, using probability proportional to size sampling, required number of sites/venues were randomly selected in each selected city/county to ensure the recruitment of required number of MSM per site.

For internet based sampling, notifications regarding the study and invitations for participation were posted in the websites of the local CBOs of MSM. Additionally, the study was introduced and promoted in the discussion forums of local gay websites and online chat rooms by posting the IRB- approved introduction material.

### Demographic and behavior measures

During each annual round of National HIV/AIDS surveillance in China, socio-demographic and behavioral information were collected from a national-level representative sample of each target population groups. Alike others, each recruited MSM were also interviewed face-to-face using structured, pre-tested questionnaires. Socio-demographic information included age, marital status, residency and educational level. To understand sexual behavioral patterns, information was collected regarding non-commercial and commercial anal sex with men as well as vaginal sex with women. Frequency of condom use during these sexual acts during last six months, condom use during last anal sex with male and consistent condom use during anal sex with male in the past month were asked to evaluate condom use patterns. Lifetime history of injecting drugs, and self-identified sexual orientation (homosexual/bisexual) were also enquired. HIV related knowledge of the participants was evaluated using eight questions while 75% correct response was defined as having adequate correct knowledge. Availability of related services were evaluated by collecting data on distribution of free condom/lubricants, needle exchange, awareness programs, peer education, treatment, counseling and testing for HIV/STIs. Subjects were also additionally enquired if they got tested for HIV in the past year, knew the testing results and received any kind of HIV related services. The interviews were completely anonymous: no identification information was collected from any subject. Each subject was provided with a unique study-specific identification number (UID).

### Serological testing

Five ml venous blood sample was collected from each participant for HIV, syphilis and HCV antibody testing using the standard protocol and laboratory methods of China NCAIDS^23^. For both HIV and HCV, samples positive for a highly sensitive Enzyme-Linked Immunosorbent Assay (ELISA) test (ELISA-1) were subjected to a highly specific ELISA (ELISA-2) to be confirmed as positive. Syphilis antibodies were screened using ELISA test and ELISA positives were confirmed by Rapid Plasma Reagin (RPR). All the screening and confirmatory tests were conducted at designated and certified laboratories in local CDC or CDC accredited hospitals. The anonymous test results were linked with the interview data through the UIDs.

### Data analysis

Collected data along with the results of the serological tests (for the detection of HIV, syphilis and HCV antibodies) were entered into the database management system of the National HIV/AIDS surveillance in China, exported from the system and were thoroughly cleaned. SAS version 9.2^24^ and SPSS version 19.0^25^ were used for all statistical analyses. Descriptive analyses were conducted to determine the distribution of the socio-demographic factors, sexual behaviors and to calculate the prevalence proportions of HIV, syphilis and HCV (among homosexuals, bisexuals and overall). We also assessed the trend of the prevalence of the selected diseases using Cochran-Armitage trend test. Bivariate and multivariate analyses were used to compare the disease epidemics and related risk behaviors of the participants in 2013 compared to 2009, while time was treated as independent variable (data from 2010–2012 were not used in the models). HIV, syphilis and HCV sero-positivity status were treated as dependent variables. In multivariate analysis, we further adjusted for age, marital status, residential area and education. ArcGIS 10.3 software (ESRI Inc., Redlands, CA, USA) was used to determine and demonstrate the geological distribution of the epidemics. Electronic maps were obtained from China CDCs.

## Results

Altogether 171,311 adult MSM were recruited, interviewed and tested under the National HIV/AIDS surveillance program in China between 2009 and 2013. The total number of participants recruited annually increased from 17,431 in 2009 to 42,680 in 2013.

### Characteristics of the study population

Overall approximately 75% participants identified themselves as homosexuals and this percentage varied considerably across years (from 71.6% in 2011 to 79.0% in 2013).(Table 1) Year-wise distribution of the socio-demographic factors among the self-identified homosexuals and bisexuals groups are also presented in Table 1. Compared to the bisexual group, homosexuals had relatively higher proportions (varied from 48.6% in 2009 to 36.5% in 2013) of younger (aged 15–24 years) and senior high school or college educated (varied from 80.0% in 2009 to 78.7% in 2013) subjects. Proportion of currently married MSM was higher (varied from 51.8% in 2009 to 64.4% in 2013) in the bisexual group. About 83.8% participants resided in the city/county from where they were recruited. Approximately 29.5% participants were recruited from pubs while another 31.0% were recruited through internet.

### HIV, syphilis and HCV prevalence and trend

During the study period, an increasing trend (from 5.5% in 2009 to 7.3% in 2013, P < 0.001) was observed in the overall HIV prevalence among Chinese MSM. Similar pattern was also observed in two self-identified subgroups, with adjusted odds ratios (aOR) of 1.33 [95% Confidence Intervals (95% CI) = 1.22–1.45] and 1.27 (95% CI = 1.07–1.50) for homosexual and bisexual participants respectively. HIV prevalence among homosexuals was consistently higher than bisexuals during the entire study period (Table 2).

**Figure 1:**
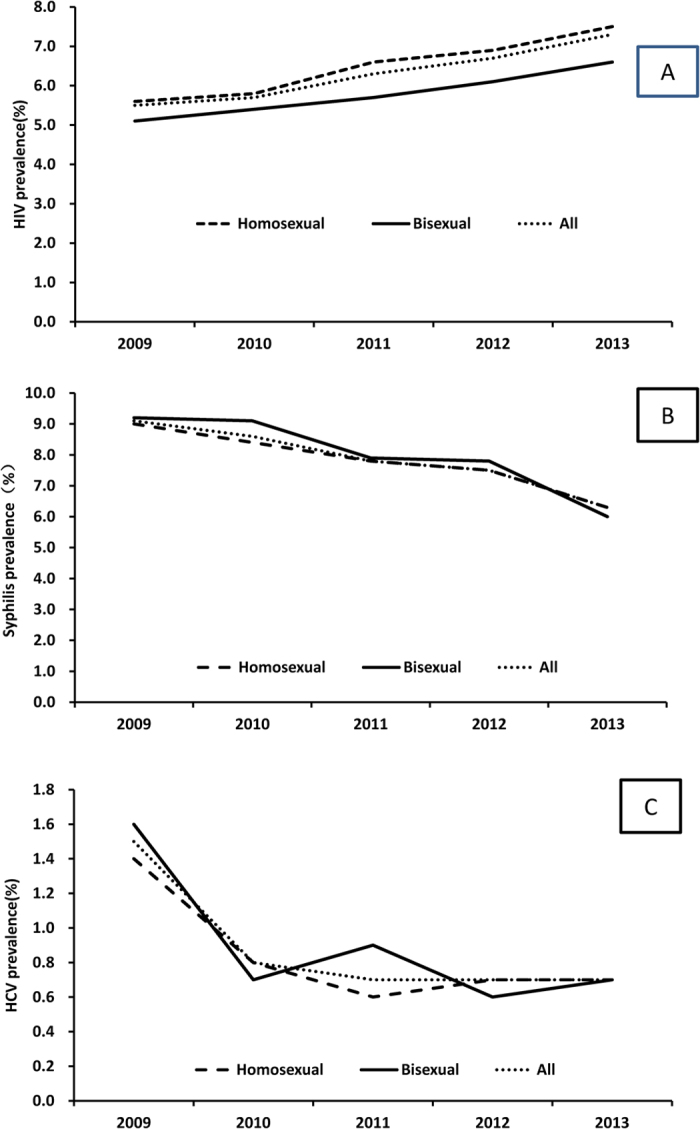
Trend of HIV (**A**), syphilis (**B**) and HCV (**C**) prevalence for the participants during 2009 and 2013 in China. (Created by Microsoft Excel, Redmond, Washington, United States, available at: https://store.office.com/en-001/appshome.aspx?ui=en-US&rs=en-001&ad=HK).

Unlike HIV, during the study period, decreasing trends were observed for syphilis (from 9.1% in 2009 to 6.3% in 2013, P < 0.001) and HCV prevalence (from1.5% in 2009 to 0.7% in 2013, P < 0.001), both overall and among subgroups (Fig. 1).

### HIV/STI related behaviors and services

Majority were engaged in anal sex with male during last six months and during the last anal sex, about 75% used condom. Use of condom increased consistently over time in both homosexual and bisexual groups, with aORs of 1.54 (95% CI = 1.47–1.62) and 1.33 (95% CI = 1.21–1.46), respectively (Table 3). Proportion of consistent condom use also increased gradually over time (from 40.4% in 2009 to 48.8% in 2013, P < 0.001).

While proportion of subjects engaged in commercial anal sex with male in last six months gradually decreased in both homosexual (aOR = 0.63, 95% CI = 0.58–0.68) and bisexual groups (aOR = 0.61 95% CI = 0.54–0.69), the proportions of condom use during the last commercial sex increased significantly.

Proportion of subjects reporting lifetime use of injectable drugs was higher among bisexuals but in both homosexuals and bisexuals these proportions decreased gradually, with aORs of 0.51 (95% CI = 0.43–0.62) and 0.32 (95% CI = 0.24–0.42), respectively.

### Correlates of HIV sero-positivity

Compared to HIV positive participants, greater proportion of HIV negative MSM used condom during the last anal intercourse. Similar results were also observed for consistent condom use during anal intercourse in the last six months. (Table 4) Lifetime use of injectable drugs was also higher among HIV sero-positive group. Proportion of participants who had better knowledge regarding HIV and who received HIV-preventive services during last year were higher among HIV negatives compared to their sero-positive counterparts. Correlates of syphilis and HCV sero-positivity were listed in supplement A and B.

### Geographic distributions

**Figure 2:**
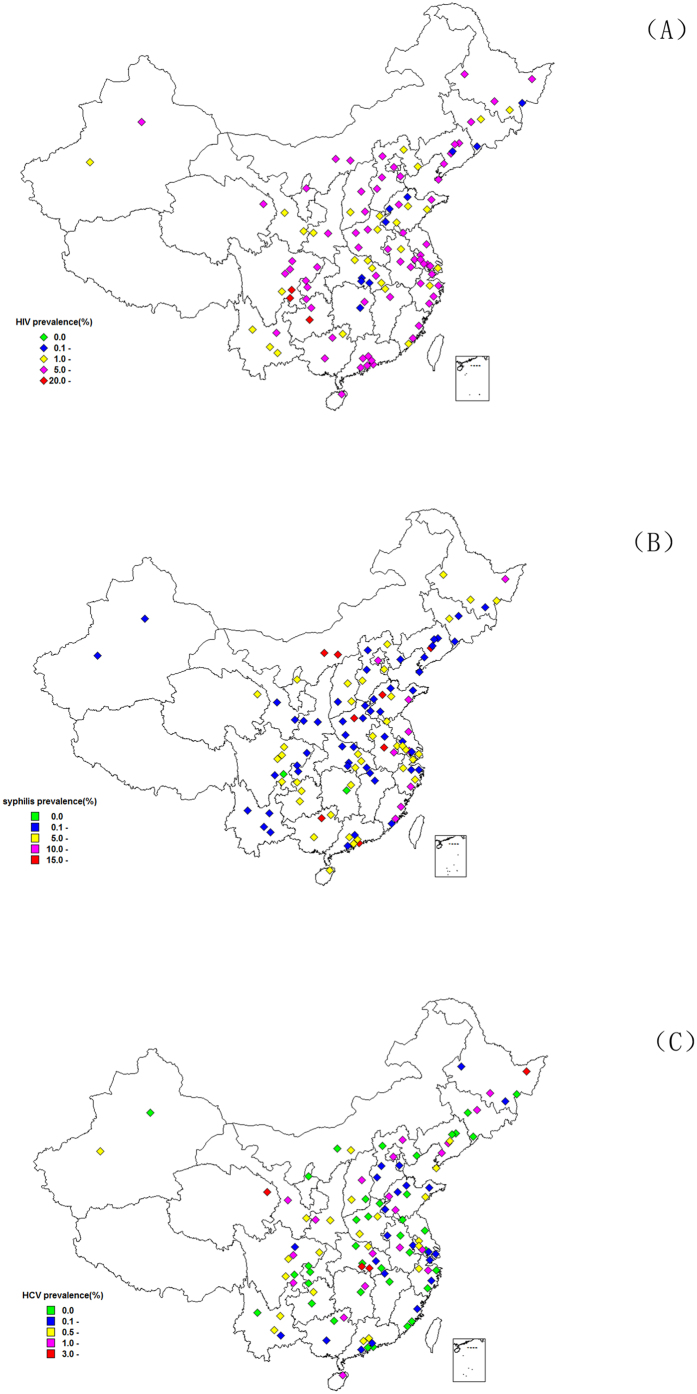
Maps of China showing the geographical distribution of HIV (**A**), syphilis (**B**), and hepatitis C virus (**C**) prevalence among MSM in 2013 in 107 sites. Abbreviations: HCV, hepatitis C virus; HIV, human immunodeficiency virus. (Created by ArcGIS 10.3 software, ESRI Inc., Redlands, CA, USA, available at: https://www.arcgis.com/home/).

Geographic distribution of HIV, syphilis and HCV sero-positivity in 107 sampling sites during 2013 is presented in Fig. 2. One site in Guizhou Province and two sites in Sichuan province had highest HIV prevalence (>20%). For syphilis, two sites in Inner Mangolia, one site each in Henan, Guangdong, Guangxi, Shandong, Anhui and Liaoning reported a prevalence of over 15%. Two sites in Hubei, one site in Helongjiang and Qinghai had HCV prevalence >3%. A significant positive correlation was observed between HIV and syphilis prevalence (r = 0.38, p = 0.03). However, HCV and syphilis were weakly correlated (r = 0.20, p = 0.27). There was no correlation between HIV and HCV prevalence (r = 0.06, p = 0.77) (Table 5).

## Discussion

Knowing the trends of infectious diseases like HIV, syphilis and HCV appeared to be critical for their control in China, as they cumulatively constituted a worrisome public health challenge, particular among MSM^6^,^7^,^10^. However, few national level studies had ever focused on the trends of these diseases^13^,^26^. This is the second paper (after FSW)^27^, in our research series that focused on the trend of HIV, syphilis and HCV prevalence among high risk populations in China where we investigated the trends of prevalence of these diseases, relevant behavioral trends and geologic distribution along with their correlations among MSM in this country.

An alarmingly high HIV prevalence was observed among Chinese MSM, particularly among the self-identified homosexuals, higher than a previous investigation among 47,231 MSM in 61 cities in China during 2008^28^ and the results of a meta-analysis conducted at 2009^29^. However, it was similar to the results of one study that integrated the results of surveillance data and systematic review^30^. Given the poor HIV related awareness among people living with HIV/AIDS^7^ and the persistently high unprotected anal intercourse among MSM in China^31^, there was a high likelihood of alarming upsurge of the epidemic within this high-risk population.

The observed HIV prevalence increased consistently during the study period, in both homosexual and bisexual groups emphasizing the fact that expanding HIV epidemic among MSM was one of the principal barriers for the success of HIV prevention programs of China^6^. Persistently high risk behaviors and lack of awareness regarding HIV among MSM in China appeared to be the major contributors^32^. Being unaware about HIV, persistent high risk sexual behaviors by HIV seropositive subjects could have resulted in further transmission of HIV^33^. Among Chinese MSM high HIV incidence and better survival of PLWHA attributed to expanding coverage of anti-retroviral therapy, could also have played important roles in the increasing trend in HIV prevalence^34^,^35^. The increasing use of internet to find partners could be another potential reason, as finding casual and multiple partners became easier through internet^36^.

Compared to homosexual participants, bisexual MSM had lower HIV prevalence, although similar risk behaviors were observed in the two groups. The reason for this disparity was unclear and warranted further studies.

The observed syphilis prevalence during 2009 corroborated with the results of a meta-analysis conducted in 2009^28^, but it was lower than the prevalence reported by the comprehensive cross-sectional study conducted in China in 2008^28^. Although the prevalence of syphilis was observed to be high among Chinese MSM, it decreased consistently with time. A major part of this reduction could be attributed to the expansion of the syphilis testing and control programs^7^,^31^. As syphilis is curable, strategies for the prevention of its transmission included early screening and appropriate treatment^37^. In the past few years, China dramatically increased the coverage of screening and treatment for both HIV and syphilis, particularly among high risk populations. Several interventions (condom promotion, behavior change, enhanced coverage for testing and treatment for HIV/STI) targeting MSM were implemented together across the country during this period^4^,^38^,^39^. These programs perhaps cumulatively minimized the burden of syphilis among MSM in China.

HCV prevalence was found to be low among Chinese MSM and during the study period a declining trend was observed. The measured prevalence was similar to the findings from a study conducted in seven cities in China^40^ but a little higher than the reported HCV burden from two studies in Beijing^13^,^14^. Compared to these studies conducted in different sites of China, the current study had larger sample size and better geographic coverage, thus the HCV epidemic scenario among Chinese MSM revealed by it was expected to be more reliable.

Corroborating one prior observation from Guangzhou^31^, this study revealed an interesting dissimilarity between the trends of occurrences of HIV and other STIs. While burden of HCV and syphilis decreased by almost 50% and 30% respectively, HIV sero-prevalence did increase by more than 30%. Increased coverage for HIV and syphilis screening and prevention programs could be the main reason for the contrasting trend between HIV and syphilis^31^ among MSM, still further research seemed necessary to clarify the underlying mechanisms. While behavioral modifications potentially minimized the incidence, cure of the disease owing to early diagnosis (through screening) and appropriate antibiotic use potentially reduced the number of existing cases of syphilis. Thus cumulatively over time, the prevalence of the disease reduced in this population. On the other hand, for HIV, although new occurrences probably decreased a bit due to some behavioral changes, owing to the increased coverage of anti-retroviral therapy, survival improved, resulting in an overall increase in the prevalence of HIV among MSM in China.

Significant decrease in sexual risk behaviors was also observed consistently among the participants during the study period. This appeared to be a positive sign considering the role of these behaviors in increasing the risk of acquisition of HIV and other STIs^9^,^41^.

Similar to our previous article that focused on FSWs^27^, large sample size, longer and continuous observation time, diversity of the sample and uniformity of the protocol were the main strengths of our study.

As an observational study, our results also had several important limitations. We did not collect information regarding non-response and our study might have suffered from some generalizability issues if the participating MSM were different from the non-participating group. Keeping the large sample size and robust sampling design in mind, we did not consider lack of generalizability to be a big issue in this study. Selection bias could also be a possibility if non-participation was affected by their HIV sero-status as well as the independent factors. But we believe that if present the magnitude of the selection bias will be potentially small, owing to the fact that across the long study time it was very less likely that both the independent and dependent variables of our study would have influenced participation consistently. Alike any other study involving snowball sampling, potential threats to generalizability were there due to the relatively higher possibility of non-representative seed recruitment. Also during snowball sampling, more co-operative subjects and those having larger personal network had potentially higher likelihood of being selected^42^. Despite these possibilities, we were probably able to restore overall representativeness of our sample by incorporating probability proportional to size method during the internet and venue based sampling in our mixed-method sampling strategy in addition to snow-balling. Still some possibility of non-recruitment of MSM from unknown venues remained. Information bias was another potential vulnerability. For example: self-identified sexual orientation might have been reported with different level of certainty by different subjects and these non-unique definitions had potentials for introducing misclassification. In these face-to-face interviews, self-reported information were very likely to suffer from social desirability bias. While some of our results could have suffered from this bias, to avoid this, we could not collect information on their role (insertive/receptive/versatile) during anal sex with another male. Another important limitation for our study is that a subject that living with HIV could be selected in multiple years, and this multiple selection could have potential impact on the overall HIV epidemic. Our system data shown that among the HIV positive cases identified during the study period, around 10% (1079/11015) were already identified before. However, we are not sure the proportion of these positive cases that were captured by the surveillance for multiple times (≥2), and not sure about its impact on overall epidemic.

## Conclusion

Prevalence of HIV was found to be high among MSM in China. Moreover, in this hard-to-reach, high risk population, HIV prevalence did show an increasing trend, while the prevalence of syphilis and HCV were declining consistently during 2008–2013. Condomless anal intercourse and injectable drug use were positively associated with HIV infection. Besides further expansion of HIV/STI testing and related services, targeted intervention programs were urgently required to control the spread of HIV within and from MSM population in China. Deeper thematic investigational dive into the issue were also called for combating the persistently high and gradually increasing HIV burden among MSM in China.

## Additional Information

**How to cite this article**: Qin, Q. *et al.* Changing trend of HIV, Syphilis and Hepatitis C among Men Who Have Sex with Men in China. *Sci. Rep.* **6**, 31081; doi: 10.1038/srep31081 (2016).

## Supplementary Material

Supplementary Information

## Figures and Tables

**Table 1:** Demographic characteristics of MSM recruited between 2009 and 2013 in China (N = 171, 311).

Variables	2009 (N = 17431)		2010 (N = 34191)		2011 (N = 37099)		2012 (N = 39910)		2013 (N = 42680)	
	homosexual %	bisexual %	homosexual %	bisexual %	homosexual %	bisexual %	homosexual %	bisexual %	homosexual %	bisexual %
Percentage of the class	77.1	22.9	74.3	25.7	71.6	28.4	75.2	24.8	79.0	21.0
Sampling site**/**methods										
* Pub, Disco, Tearoom, or Club*	35.9	36.1	33.0	33.4	27.7	30.7	28.5	28.6	26.5	22.7
* Spa or bathhouse, Sauna or Massage*	11.1	14.0	11.0	15.4	12.7	17.2	11.2	15.2	9.8	11.7
* Park, Public Restroom, or Public Lawn*	11.6	17.8	9.5	11.3	7.9	9.4	11.1	12.9	8.2	13.7
* Internet*	27.0	20.3	26.2	21.7	31.9	22.4	31.7	25.9	35.1	27.9
* Others*										
	13.1	10.9	18.8	16.9	19.2	20.0	17.3	17.1	20.1	23.8
Age										
* 15*–*24*	48.6	26.0	45.7	21.9	44.1	18.8	40.2	17.4	36.5	13.3
* 25–49*	49.3	71.1	52.0	74.4	53.1	77.2	56.8	78.1	60.0	81.6
* 50−*	2.1	2.9	2.3	3.8	2.8	3.9	3.0	4.5	3.5	5.1
Marital status										
* Never Married and not cohabited*	82.0	41.8	79.6	36.6	78.1	31.2	77.9	30.8	76.5	29.9
* Married*	10.4	51.8	13.2	57.3	14.3	61.9	14.0	63.2	14.6	64.4
* Never married but cohabited*	1.4	2.7	1.0	2.8	0.8	3.3	1.2	2.2	1.5	2.2
* Divorced or widowed*	6.0	3.5	5.9	3.0	6.8	3.5	6.7	3.7	7.4	3.4
Resident										
* The sampling province*	84.4	83.2	82.0	81.0	83.2	85.1	84.3	84.5	85.1	84.5
* Other province*	15.5	16.5	17.6	18.6	16.6	14.8	15.5	15.4	14.8	15.4
Education										
* Illiteracy or only attended elementary*	2.5	4.6	2.5	4.0	2.8	4.3	2.8	4.3	2.8	4.4
* Attended Junior high school*	17.4	24.0	17.3	23.8	17.4	22.5	16.8	23.5	18.4	23.7
* Attended Senior high school or above*	80.0	71.2	80.0	72.0	79.7	73.1	80.4	72.2	78.7	71.8

**Table 2:** HIV, syphilis and HCV prevalence for the participants during 2009 and 2013 in China (N = 171, 311).

Variables	2009 (%)	2010 (%)	2011 (%)	2012 (%)	2013 (%)	P for Trend	Crude OR (95% CI)		Adjusted OR* (95% CI)		***P*** Value for adjusted model
HIV											
Homosexual	5.6	5.8	6.6	6.9	7.5	<0.001	1.38	(1.26–1.5)	1.33	(1.22–1.45)	<0.001
Bisexual	5.1	5.4	5.7	6.1	6.6	<0.001	1.31	(1.11–1.54)	1.27	(1.07–1.5)	0.01
All	5.5	5.7	6.3	6.7	7.3	<0.001					
Syphilis											
Homosexual	9.0	8.4	7.8	7.5	6.3	<0.001	0.68	(0.63–0.73)	0.64	(0.60–0.69)	<0.001
Bisexual	9.2	9.1	7.9	7.8	6.0	<0.001	0.63	(0.55–0.72)	0.58	(0.51–0.67)	<0.001
All	9.1	8.6	7.8	7.5	6.3	<0.001					
HCV											
Homosexual	1.4	0.8	0.6	0.7	0.7	<0.001	0.45	(0.37–0.56)	0.44	(0.35–0.54)	<0.001
Bisexual	1.6	0.7	0.9	0.6	0.7	<0.001	0.46	(0.32–0.65)	0.43	(0.3–0.63)	<0.001
All	1.5	0.8	0.7	0.7	0.7	<0.001					

^*^Multivariate models were adjusted for the following variables: age, marital status, resident and education.

**Table 3:** HIV related knowledge, services and behaviors for MSM during 2009 and 2013 in China.

Variables	2009 (%)	2010 (%)	2011 (%)	2012 (%)	2013 (%)	P for Trend	Crude OR (95% CI)		Adjusted OR* (95% CI)		P for Adjusted Model
Engaged in anal sex in the last 6 months											
Homosexual	88.85	83.45	86.37	87.27	86.74	<0.001	0.82	(0.77–0.87)	0.83	(0.78–0.88)	<0.001
Bisexual	85.31	82.97	83.05	85.20	85.08	0.003	0.98	(0.88–1.09)	0.97	(0.87–1.08)	0.55
All	88.04	83.33	85.42	86.75	86.39	<0.001					
Used condom during last anal intercourse with male											
Homosexual	71.01	73.41	74.35	75.50	78.81	<0.001	1.52	(1.45–1.59)	1.54	(1.47–1.62)	<0.001
Bisexual	73.27	73.78	73.57	77.11	77.59	<0.001	1.26	(1.15–1.39)	1.33	(1.21–1.46)	<0.001
All	71.51	73.51	74.13	75.89	78.55	<0.001					
Consistent used condom during anal sex in the last 6 months											
Homosexual	39.89	42.95	43.47	47.00	49.25	<0.001	1.46	(1.4–1.53)	1.47	(1.41–1.54)	<0.001
Bisexual	42.27	41.53	42.80	45.86	46.95	<0.001	1.21	(1.11–1.31)	1.27	(1.16–1.38)	<0.001
All	40.41	42.59	43.29	46.72	48.77	<0.001					
Engaged in commercial anal sex with male in the last 6 months											
Homosexual	10.34	9.3	7.46	7.86	6.46	<0.001	0.6	(0.56–0.65)	0.63	(0.58–0.68)	<0.001
Bisexual	17.23	14.5	10.08	11.74	9.39	<0.001	0.5	(0.44–0.56)	0.61	(0.54–0.69)	<0.001
All	11.87	10.62	8.18	8.8	7.07	<0.001					
Consistent used condom during commercial anal sex in the last 6 months											
Homosexual	46.06	51.91	51.51	57.2	56.86	<0.001	1.54	(1.33–1.79)	1.53	(1.32–1.78)	<0.001
Bisexual	47.54	52.56	55.63	46.88	50.21	0.51	1.11	(0.89–1.39)	1.18	(0.93–1.5)	0.17
All	46.54	52.14	52.91	53.85	55.03	<0.001					
Used condom at last commercial anal intercourse											
Homosexual	73.13	77.34	75.10	83.07	83.72	<0.001	1.89	(1.58–2.25)	2.03	(1.69–2.43)	<0.001
Bisexual	75.3	77.96	77.96	84.32	77.95	0.01	1.16	(0.89–1.5)	1.26	(0.95–1.66)	<0.001
All	73.83	77.55	76.07	83.48	82.12	<0.001					
Used drug in lifetime											
Homosexual	1.47	0.86	0.92	0.78	0.74	<0.001	0.5	(0.42–0.61)	0.51	(0.43–0.62)	<0.001
Bisexual	3.6	1.64	1.84	1.23	0.95	<0.001	0.26	(0.2–0.34)	0.32	(0.24–0.42)	<0.001
All	1.95	1.06	1.18	0.89	0.79	<0.001					

^*^Multivariate models were adjusted for the following variables: age, marital status, resident and education.

**Table 4:** HIV related knowledge, services and behaviors for HIV negative and positive MSMs in China (2009–2013).

Variables	2009		2010		2011		2012		2013	
Engaged in anal sex in the last 6 months										
HIV−	88.1	(13999/15891)	83.4	(26376/31644)^a^	85.5	(29570/34598)	86.8	(32210/37122)	86.1	(33953/39431)^a^
HIV+	88.9	(816/918)	85.3	(1632/1913)	85.6	(1994/2330)	86.4	(2304/2666)	90.0	(2796/3107)
Used condom during at last anal intercourse with male										
HIV−	71.7	(9925/13844)	73.9	(19379/26209)^a^	74.7	(21910/29337)^a^	76.4	(24502/32083)^a^	79.3	(26800/33782)^a^
HIV+	69.6	(562/808)	67.2	(1089/1620)	66.6	(1323/1986)	68.5	(1573/2295)	68.9	(1922/2788)
Consistent used condom during anal sex in the last 6 months										
HIV−	40.2	(5566/13848)^a^	43.1	(11327/26263)^a^	44.0	(12922/29381)^a^	47.6	(15267/32100)^a^	49.7	(16825/33880)^a^
HIV+	35.9	(289/804)	33.5	(545/1625)	33.8	(671/1985)	35.2	(807/2294)	37.6	(1050/2791)
Engaged in commercial anal sex with male in the last 6 months										
HIV−	12.0	(1683/13981)	10.6	(2800/26446)	8.2	(2426/29549)	8.9	(2854/32176)^a^	7.1	(2399/33939)^a^
HIV+	11.3	(92/815)	11.5	(187/1633)	7.8	(156/1995)	7.6	(175/2298)	6.9	(192/2795)
Consistent used condom during commercial anal sex in the last 6 months										
HIV−	47.0	(775/1649)	52.7	(1477/2803)^a^	53.0	(1273/2400)	55.3	(1562/2825)^a^	54.9	(1308/2381)
HIV+	40.9	(36/88)	42.1	(80/190)	50.6	(78/154)	27.4	(48/175)	55.3	(105/190)
Used condom during last commercial anal intercourse										
HIV−	74.7	(1242/1663)^a^	78.0	(2199/2818)^a^	76.1	(1834/2410)	84.3	(2386/2832)^a^	82.7	(1965/2376)^a^
HIV+	67.8	(61/90)	70.4	(133/189)	75.2	(115/153)	70.1	(122/174)	74.7	(142/190)
Used drug in lifetime										
HIV−	1.9	(296/15708)	1.0	(329/31736)	1.1	(390/34539)^a^	0.9	(319/37067)^a^	0.7	(289/39401)^a^
HIV+	2.6	(24/908)	1.1	(22/1915)	2.0	(47/2326)	1.3	(35/2660)	1.5	(46/3101)
Correct HIV related knowledge										
HIV−	86.7	(13819/15935)	90.3	(28785/31880)^a^	91.2	(31574/34631)^a^	92.8	(34450/37141)^a^	93.9	(37028/39433)^a^
HIV+	87.5	(805/920)	87.9	(1694/1927)	89.0	(2080/2336)	90.5	(2413/2666)	92.1	(2863/3107)
Being received any kind of HIV related services in last year										
HIV−	77.9	(12410/15922)	79.6	(25358/31845)^a^	80.7	(27936/34614)^a^	81.5	(30248/37134)^a^	81.7	(32220/39428)^a^
HIV+	75.8	(697/920)	77.4	(1487/1922)	73.2	(1709/2334)	73.5	(1959/2666)	77.6	(2411/3106)

^a^represents statistical significance.

**Table 5:** Correlation between HIV and syphilis, HIV and HCV, HCV and syphilis among MSM in different regions of China, 2013.

Region*	HIV and syphilis		HIV and HCV		HCV and syphilis	
	Correlation (r)	P	Correlation (r)	P	Correlation (r)	P
East	0.64	0.12	−0.21	0.64	0.04	0.94
Northeast	−0.5	0.67	0.50	0.67	0.50	0.67
North	0.00	1.00	0.60	0.28	−0.20	0.75
South Central	0.6	0.21	−0.31	0.54	−0.09	0.87
Southwest	0.7	0.19	0.05	0.94	0.67	0.22
Northwest	0.9	0.04	0.10	0.87	0.00	1.00
Overall	0.38	0.03	0.06	0.77	0.20	0.27

^*^East China: Anhui, Fujian, Jiangsu, Jiangxi, Shandong, Shanghai, Zhejiang; Northeast China: Heilongjiang, Jilin, Liaoning; North China: Beijing, Hebei, Inner Mongolia, Shanxi, Tianjin; South Central China: Guangdong, Guangxi, Hainan, Henan, Hubei, Hunan; Northwest China: Gansu, Ningxia, Qinghai, Shaanxi, Xinjiang; Southwest China: Chongqing, Guizhou, Sichuan, Tibet, Yunnan.
