# Supplementary material for: Changing trend of HIV, Syphilis and Hepatitis C among Men Who Have Sex with Men in China
Source: Sci Rep. 2016 Aug 18;6:31081. doi: 10.1038/srep31081 (PMC4989164; doi:10.1038/srep31081)
Supplement: Supplementary Information [file srep31081-s1.docx]

**Changing trend of HIV, Syphilis and Hepatitis C among Men Who Have Sex with Men in China**

Qianqian Qin^1‡^, Weiming Tang^2,3‡^, Lin Ge^1^, Dongmin Li^1^, Tanmay Mahapatra^4^, Liyan Wang^1^, Wei Guo^1^, Yan Cui^1,*^, Jiangping Sun^1,*^

^1^ *National Center for AIDS/STD Control and Prevention, Chinese Center for Disease Control and Prevention, Beijing, China; ^2^ University of North Carolina at Chapel Hill, Project-China. No. 2 Lujing Road, Guangzhou, 510095, China;^3^School of Medicine, University of North Carolina at Chapel Hill, Chapel Hill, NC, 27514, USA; ^4^National Institute of Cholera and Enteric Diseases, Kolkata, 700010, India.*

**Supplement A，HIV related knowledge, services and behaviors for syphilis negative and positive MSMs in China (2009-2013)**

| **Variables** | **2009** |  | **2010** |  | **2011** |  | **2012** |  | **2013** | |
| --- | --- | --- | --- | --- | --- | --- | --- | --- | --- | --- |
| **Engaged in anal sex in the last 6 months** | | | | | | | | | | |
| syphilis- | 88.1 | (13402/15213) | 83.4 | (25539/30615) | 85.5 | (29096/34034) | 86.7 | (31891/36784) | 86.3 | (34413/39879)^a^ |
| syphilis+ | 89.5 | (1358/1518) | 84.1 | (2431/2890) | 85.3 | (2461/2884) | 87.3 | (2622/3003) | 87.9 | (2338/2661) |
| **Used condom during at last anal intercourse with male** | | | | | | | | | | |
| syphilis- | 71.5 | (9480/13257) | 73.5 | (18661/25382) | 74.5 | (21495/28862)^a^ | 76.2 | (24207/31761)^a^ | 78.8 | (26968/34244)^a^ |
| syphilis+ | 71.6 | (961/1342) | 73.6 | (1774/2409) | 70.7 | (1734/2454) | 71.4 | (1867/2616) | 75.4 | (1756/2328) |
| **Consistent used condom during anal sex in the last 6 months** | | | | | | | | | | |
| syphilis- | 39.8 | (5271/13257) | 42.6 | (10829/25431) | 43.7 | (12629/28909)^a^ | 47.2 | (14989/31778)^a^ | 49.0 | (16819/34341)^a^ |
| syphilis+ | 41.1 | (552/1342) | 42.5 | (1027/2419) | 39.2 | (960/2450) | 41.5 | (1084/2615) | 45.4 | (1058/2331) |
| **Engaged in commercial anal sex with male in the last 6 months** | | | | | | | | | | |
| syphilis- | 12.0 | (1600/13383) | 10.5 | (2700/25601) | 8.1 | (2366/29077) | 8.8 | (2791/31852) | 7.1 | (2438/34398) |
| syphilis+ | 12.5 | (170/1358) | 11.8 | (288/2440) | 8.7 | (214/2460) | 9.1 | (238/2621) | 6.5 | (152/2337) |
| **Consistent used condom during commercial anal sex in the last 6 months** | | | | | | | | | | |
| syphilis- | 46.9 | (735/1566) | 52.2 | (1411/2705) | 53.3 | (1247/2340) | 54.4 | (1503/2764)^a^ | 55.0 | (1332/2421) |
| syphilis+ | 44.0 | (73/166) | 50.9 | (147/289) | 49.1 | (104/212) | 45.3 | (107/236) | 53.7 | (80/149) |
| **Used condom during last commercial anal intercourse** | | | | | | | | | | |
| syphilis- | 74.9 | (1186/1583) | 77.2 | (2100/2720) | 75.9 | (1784/2350) | 84.0 | (2326/2769)^a^ | 82.6 | (1994/2415)^a^ |
| syphilis+ | 68.1 | (113/166) | 80.9 | (233/288) | 78.2 | (165/211) | 76.8 | (182/237) | 74.7 | (112/150) |
| **Used drug in lifetime** | | | | | | | | | | |
| syphilis- | 1.9 | (279/15046)^a^ | 1.0 | (318/30700) | 1.2 | (402/33975) | 0.9 | (319/36726) | 0.7 | (296/39849)^a^ |
| syphilis+ | 2.6 | (39/1493) | 1.1 | (32/2898) | 1.2 | (35/2880) | 1.2 | (35/3000) | 1.5 | (39/2655) |
| **Correct HIV related knowledge** | | | | | | | | | | |
| syphilis- | 86.9 | (13253/15256) | 90.3 | (27846/30848)^a^ | 91.3 | (31102/34071)^a^ | 92.7 | (34101/36801) | 93.8 | (37423/39881)^a^ |
| syphilis+ | 85.5 | (1301/1521) | 89.0 | (2587/2907) | 88.1 | (2543/2886) | 91.9 | (2761/3005) | 92.8 | (2470/2661) |
| **Being received any kind of HIV related services in last year** | | | | | | | | | | |
| syphilis- | 77.6 | (11828/15245)^a^ | 79.4 | (24474/30815) | 80.3 | (27336/34057) | 81.0 | (29819/36794)^a^ | 81.5 | (32501/39875) |
| syphilis+ | 80.5 | (1223/1519) | 80.1 | (2324/2900) | 79.8 | (2300/2881) | 79.4 | (2387/3005) | 80.1 | (2132/2661) |

**Note: ^a^ represents statistical significance.**

**Supplement B，HIV related knowledge, services and behaviors for HCV negative and positive MSMs in China (2009-2013)**

| **Variables** | **2009** |  | **2010** |  | **2011** |  | **2012** |  | **2013** | |
| --- | --- | --- | --- | --- | --- | --- | --- | --- | --- | --- |
| **Engaged in anal sex in the last 6 months** | | | | | | | | | | |
| HCV- | 87.9 | (12506/14234) | 83.5 | (27722/33214) | 85.5 | (31325/36651) | 86.7 | (34268/39508) | 86.4 | (36501/42249) |
| HCV+ | 90.1 | (191/212) | 84.6 | (225/266) | 87.5 | (232/265) | 88.3 | (233/264) | 85.7 | (245/286) |
| **Used condom during at last anal intercourse with male** | | | | | | | | | | |
| HCV- | 70.3 | (8710/12382) | 73.5 | (20256/27547) | 74.2 | (23060/31086) | 75.8 | (25889/34132) | 78.6 | (28547/36323) ^a^ |
| HCV+ | 63.8 | (120/188) | 70.9 | (158/223) | 73.5 | (169/230) | 77.3 | (180/233) | 71.3 | (174/244) |
| **Consistent used condom during anal sex in the last 6 months** | | | | | | | | | | |
| HCV- | 39.9 | (4932/12367)^a^ | 42.5 | (11741/27603) | 43.3 | (13480/31129) | 46.8 | (15966/34148) | 48.8 | (17764/36422) |
| HCV+ | 30.9 | (58/188) | 45.5 | (102/224) | 47.4 | (109/230) | 45.1 | (105/233) | 46.1 | (113/245) |
| **Engaged in commercial anal sex with male in the last 6 months** | | | | | | | | | | |
| HCV- | 12.7 | (1581/12479) ^a^ | 10.7 | (2966/27794) | 8.2 | (2558/31305) | 8.8 | (2997/34228) ^a^ | 7.0 | (2569/36485) |
| HCV+ | 17.7 | (34/192) | 9.8 | (22/224) | 9.5 | (22/232) | 13.7 | (32/233) | 9.0 | (22/245) |
| **Consistent used condom during commercial anal sex in the last 6 months** | | | | | | | | | | |
| HCV- | 45.8 | (710/1550) | 52.1 | (1548/2973) | 53.0 | (1341/2530) | 53.8 | (1597/2970) | 55.0 | (1403/2549) |
| HCV+ | 36.7 | (11/30) | 47.6 | (10/21) | 45.5 | (10/22) | 43.3 | (13/30) | 45.5 | (10/22) |
| **Used condom during last commercial anal intercourse** | | | | | | | | | | |
| HCV- | 74.5 | (1164/1562) ^a^ | 77.6 | (2318/2987) | 76.1 | (1932/2539) | 83.4 | (2482/2975) | 82.0 | (2087/2544) |
| HCV+ | 53.1 | (17/32) | 71.4 | (15/21) | 77.3 | (17/22) | 83.9 | (26/31) | 90.9 | (20/22) |
| **Used drug in lifetime** | | | | | | | | | | |
| HCV- | 2.1 | (299/14047) ^a^ | 1.0 | (337/33308) ^a^ | 1.1 | (411/36588) ^a^ | 0.8 | (331/39447) ^a^ | 0.8 | (331/42213) |
| HCV+ | 4.3 | (9/208) | 4.9 | (13/265) | 9.8 | (26/265) | 8.7 | (23/264) | 1.4 | (4/286) |
| **Correct HIV related knowledge** | | | | | | | | | | |
| HCV- | 86.2 | (12289/14262) ^a^ | 90.2 | (30177/33463) | 91.1 | (33410/36690) | 92.6 | (36611/39527) | 93.8 | (39628/42251) ^a^ |
| HCV+ | 79.8 | (170/213) | 86.9 | (232/267) | 87.9 | (233/265) | 89.4 | (236/264) | 90.9 | (260/286) |
| **Being received any kind of HIV related services in last year** | | | | | | | | | | |
| HCV- | 75.8 | (10799/14251) | 79.5 | (26573/33424) | 80.2 | (29414/36671) | 80.9 | (31981/39520) | 81.4 | (34393/42245) |
| HCV+ | 77.0 | (164/213) | 78.2 | (208/266) | 83.4 | (221/265) | 82.2 | (217/264) | 83.9 | (240/286) |

**Note: ^a^ represents statistical significance.**
